# Two Cases of Angina Pectoris Following Sepsis
1A Paper read to the Bath and Bristol Branch of the British Medical Association, October 26th, 1927.


**Published:** 1927

**Authors:** Newman Neild

**Affiliations:** Senior Physician, Bristol General Hospital


### TWO CASES OF ANGINA PECTORIS FOLLOWING SEPSIS.[1]

BY

Newman Neild, M.B. Vict., M.R.C.P. Lond.,
*Senior Physician, Bristol General Hospital.*

---

Two cases which have recently come under my observation seem to favour the view that severe sepsis may predispose to angina pectoris.

The first case was that of a professional man who had lived a very healthy life, simple in his diet and abstemious both as regards alcohol and tobacco. His children are all grown up and healthy, and syphilis may be excluded as certainly as it ever can be without Wassermann tests. The one possible factor present known to play a part in the ætiology of angina pectoris was that he had many and prolonged worries.

In the early part of 1922, when he was just under 60 years of age, he was accustomed to walk up steep paths on a hill-side without breathlessness or unpleasant fatigue. Later in this year he developed a boil upon his lip causing enormous swelling of the face, so that one eye was closed. This took some time to heal, and he was very weak afterwards. He next had a septic finger, and this lasted a few weeks whilst he tried to continue his professional work. Hardly recovered from this, he developed a severe sepsis within a tight phimosis. I was asked to see him after he had had the condition for about three weeks, during which time his doctor had slit up the prepuce. The patient was in a very toxic condition, febrile, anæmic, weak, and had lost flesh considerably. Convalescence was slow. I saw him again about six weeks afterwards, as he complained of discomfort in the cardiac region with a peculiar sensation in the left arm. His pulse was soft, regular and of medium volume, with no thickening of the artery walls to be detected by the finger, and the systolic blood pressure was

[1] A Paper read to the Bath and Bristol Branch of the British Medical Association, October 26th, 1927.

slightly over 130 mm. Hg. On first examining the heart it was two finger-breadths outside the nipple line, but after examining the lungs and returning to the heart the left border was in the nipple line and the heart beat had slowed down to normal. After this he was much better for some months.

In April, 1924, he changed his residence. In June he had just attended a meeting in which he had to take an active part, and he had his first severe attack of angina. His family were thoroughly frightened by the severity of the attack and his condition after it. I again saw him soon after this. His pulse, blood pressure and heart were much as before, but the enlargement of the heart under examination was accompanied by irregularity of the beat and was slower in subsiding, nor did it quite come back to the nipple line. Under treatment he was much better for a month or so, but found that he had to move with great caution and avoid any emotion. In August he had another attack and died in a few minutes.

The second case was that of a carpenter, still living, now aged 43. He had been in the Navy for ten years. Except for a fracture of a collar-bone he had been quite healthy.

In January last he had a suppurating olecranon bursa opened in the casualty department at the General Hospital. Next day his temperature was just under 102° F., the hand and arm very red and swollen, and he was admitted under Mr. C. A. Moore. Four incisions had to be made in the left arm and forearm. The organism present was a streptococcus. The temperature remained normal or subnormal after four days, but the swelling had not entirely subsided until the tenth day. He was an in-patient for a fortnight, and when he left one incision was still discharging. He states that he was attending for some six weeks or more as an out-patient, and that he has only worked for one month since the septic infection occurred. He came under my care in June, and the following history was obtained.

A fortnight before admission he developed pneumonia, with very high temperature and delirium for several days. During this time severe vomiting attacks came on, and for four days he could retain no food. With the fall of the temperature the vomiting stopped. Four days before admission he vomited a clot of brown-coloured blood "the size of a kidney." This was followed by melæna. This was his first hemorrhage, although he had been subject to abdominal pains for a long time, nor had he vomited before having the pneumonia. He had pain whether he took food or not, but food aggravated the pain. The right lung was not yet quite normal. The heart was normal in size and there were no murmurs. No visible impulse, the pulse soft, regular and no thickening of the artery. Heart sounds, distant.

He was given the Lenhartz diet, interrupted on the sixth day for examination of the fæces for occult blood (negative) and for an X-ray examination of the stomach. Dr. Bergin's report is as follows : " *Stomach.*—Whole lower half of stomach in constant deep spasm—will not fill out. Point of maximum tenderness near pyloric canal. *Duodenum.*—Hangs down and is in spasm, tender to palpation. Meal being evacuated rapidly." Ten days later he reported : " *Stomach.*—Much less irritable than on former examination. Peristalsis regular and even, not excessive. No spasm. *Duodenum.*—Acting normally." The patient was in hospital for one month, and left after being up and about for two or three days and without pain.

He was again admitted on September 29th for anginal attacks. Five weeks before admission he woke up in the morning with a severe pain over the heart. This lasted ten minutes. A fortnight later he had a similar attack, and three attacks during the week. A few very slight attacks occurred during the following week. On the 21st he had a bad attack, during which the pain went down the left arm ; went to work but collapsed on the way. His doctor sent him to me on the 26th, there having been a severe attack lasting three-quarters of an hour.

The notes taken on admission say that " he now has no abdominal pain and the tongue is clean. Lies propped up in bed, as lying down brings on the pain. The heart on examination was as when last in hospital, and the pulse also. He says that any exertion or excitement brings on the pain. This extends down to the two inner fingers of left hand." He had two attacks following a medical examination on the 3rd of October. In one of these attacks he was thought to be dying. On the 4th he had several rather bad attacks, but felt quite well next day. He continued to have attacks, though less severe, until he was given theobromine 10 grains thrice daily, since when he has had no attacks of any kind, but any slight exertion still brings on obvious dyspnœa. A further X-ray examination showed no dilatation of the lower end of the œsophagus.

Dr. Coombs, since then, has been good enough to examine him, and confirms the absence of cardiac signs of disease when the patient is at rest. Dr. Herapath most kindly examined the patient with the electrocardiograph, and reports that the electrocardiograms are normal.

My reason for bringing forward these two cases has been to suggest that severe sepsis may predispose to angina pectoris. These two cases are, of course, insufficient evidence to prove any causal relationship. The first case occurred in a man of 62, who had lived a full life of great responsibilities with their accompanying anxieties and worries. The second patient had pneumonia, and probably gastric ulcer also, and I do not consider that a blood Wassermann test is sufficiently conclusive evidence to exclude syphilis absolutely.

In the new edition of his text-book, F. W. Price[1] puts amongst the predisposing causes heredity, the male sex, mental wear and tear, gout, syphilis, rheumatism, some of the acute infectious diseases, especially influenza and enteric, alcohol, and perhaps tea, coffee and tobacco. Arterial disease predisposes and is present in the majority of cases, and in many there is an abnormally raised blood pressure.

I do not know whether the sepsis in the bursa had anything to do with the occupation of the second patient, but should sepsis come to be a recognised cause of angina pectoris, then the medico-legal importance of this connection is obvious. Whilst I recognise that " angina pectoris gravior " is but the name of a fairly well-defined symptom occurring in more than one variety of heart disease, it is, in the great majority of cases, a symptom of cardiac conditions incapacitating a man for work.

[1] F. W. Price (Ed.), *A text-book of the Practice of Medicine*, Ed. 2, 1926, p. 887.

